# Technocrats vs. tipping points: How East Asian governance shapes global emissions

**DOI:** 10.1371/journal.pone.0339968

**Published:** 2026-01-20

**Authors:** Joseph Lavallee, Bruno Di Giusto

**Affiliations:** 1 International Business and Trade Program, International College, Ming Chuan University, Taipei, Taiwan; 2 Journalism and Mass Communication Program, International College, Ming Chuan University, Taipei, Taiwan; University of Pretoria, SOUTH AFRICA

## Abstract

Why do East Asian economies—China, Japan, South Korea, and Taiwan—lag behind expectations in climate mitigation despite their technological prowess and economic strength? To approach this question, we extend Lamb and Minx’s “architectures of constraint” framework through an integration of civic engagement and political representativeness metrics. Using principal component and cluster analyses across 28 countries responsible for 70% of global carbon emissions, we uncover six distinct climate policy architectures, notably identifying a unique East Asian developmental state cluster. This cluster features powerful bureaucracies and state-business alliances but weak civic-participatory channels, creating institutional bottlenecks that prevent public environmental concern from translating into robust climate policies. Challenging the prevailing view that democratization alone ensures ambitious climate action, our study suggests that the channels through which citizens influence policy are closely linked to climate outcomes. Given East Asia’s enormous share of global emissions, our findings have urgent implications for international climate governance strategies. To accelerate the global energy transition, tailored strategies that address East Asia’s distinct institutional dynamics are critical.

## Introduction

The window for keeping global warming below 1.5°C is closing fast: at current trends the remaining carbon budget could be spent within five years [1,2]. Because East Asia now emits 36% of the world’s CO₂—more than North America and the EU combined [3]—its policy choices will largely decide how soon that threshold will be breached and whether the 2°C threshold will follow.

Within East Asia, China is responsible for two-thirds of CO₂ emissions. However, even the advanced democracies of Japan, South Korea and Taiwan emit substantial volumes, challenging conventional assumptions about democracy and environmental policy. While earlier scholarship posited a correlation between democracy and environmental protection [4,5], many democracies struggle with climate action despite having the capacity and public support for ambitious policies. Given East Asia’s contribution to global emissions, understanding why democratic institutions fail to deliver climate action is critical. If conventional Western approaches prove ineffective in East Asia, then global climate strategies must be reconsidered.

Lamb and Minx [6] developed an innovative framework to better grasp the intricacies of the global energy transition through their concept of ‘architectures of constraint.’ Rather than focusing on isolated variables, such as democracy indexes, they examine how broader configurations of interlocking interests, institutions, and ideas create distinct ‘architectures’ that either enable or constrain climate action. Their empirical analysis identified five distinct country-clusters, revealing that political-economic structures shape climate outcomes in complex, non-linear ways. However, studies using this framework [6,7] still tend to compress important variation within democratic systems. For instance, Lamb & Minx’s ‘Wealthy OECD’ cluster contains climate leaders like Denmark alongside laggards like Australia. This suggests that finer-grained analysis is needed to capture the mechanisms through which different types of democracies—and non-democracies—approach climate governance.

The East Asian cases illustrate this need for greater specificity. Despite their different political systems—China’s autocracy versus the democracies of Japan, South Korea, and Taiwan—these countries share a distinctive governance framework: the developmental state model. Introduced by Johnson [8], Amsden [9], and Wade [10] to explain rapid industrialization in the region, this model describes states that actively orchestrate economic development through strategic planning, autonomous bureaucracies, extensive state-business coordination, and limitations on civic input. These features potentially shape how these countries approach climate governance.

Thus, to provide greater analytical depth, we seek to extend Lamb and Minx’s framework by incorporating more comprehensive measures. Specifically, we add survey-based indicators of civic engagement (NGO membership, petitions, donations, protest) and party representativeness from the 2020 International Social Survey Programme (ISSP) on the Environment [11]. These variables allow us to move beyond basic democracy scores to examine how environmental ideas flow—or fail to flow—from citizens to policy. Embedding these variables in a principal-components/cluster workflow lets us probe whether East Asia’s energy transition is shaped by a distinct constraint architecture.

We also refine the *outcome* side of the analysis. Whereas Lamb & Minx [6] gauged national performance mainly by counting climate-related laws, fossil-fuel subsidies and absolute emissions, we adopt four subscores from the Climate Change Performance Index (CCPI) [12] that jointly track not only legislative ambition but also implementation and real-world progress. Using these richer dependent variables lets us test whether distinct ‘architectures of constraint’ correspond with different mitigation trajectories.

Thus, this study asks whether metrics of civic participation and party responsiveness reveal East Asian countries as a distinct cluster in climate governance, and if so, what this institutional configuration means for the global energy transition.

## Literature review

The concept of “architectures of constraint” [6] provides a valuable framework for understanding the barriers hindering the energy transition. Drawing on Hall’s tripartite model of interests, institutions, and ideas [13], the framework conceptualizes national climate inaction as the result of interlocking economic, political, and ideational forces that become mutually reinforcing over time. Rather than treating each constraint in isolation, the framework emphasizes how these elements coalesce into stable regime types, with distinct implications for policy ambition, design, and implementation.

In this framework, interests refer to the economic stakes that different groups have in climate policy – from fossil fuel and heavy industries facing transition costs, to clean energy sectors that benefit from climate action. Institutions encompass the formal and informal rules governing political decision-making, including constitutional arrangements, electoral systems, policy-making procedures, and regulatory frameworks that enable or constrain climate action. Ideas include worldviews, public awareness, issue salience, and dominant discourses, such as ecological modernism or developmentalism. These three dimensions interact dynamically. For example, fossil fuel lobbying may secure favorable subsidy rules, which, by creating jobs and economic activity, then legitimize narratives about the economic risks of a rapid energy transition. Over time, this fusion of interests, institutions, and ideas stabilizes into a distinct ‘architecture of constraint’ that resists change.

In their empirical analysis, Lamb and Minx [6] identified five distinct country clusters with unique constraint profiles. Of these, two are of critical importance for the energy transition. First, the countries in their “Coal-Dependent Development States” cluster, representing 47% of global emissions and encompassing nations like China, India, Turkey, South Africa, and Russia, share a reliance on coal, a developmental focus, and challenges dealing with corruption and governance capacity. Second, countries in their “Wealthy OECD Nations” cluster, responsible for 30% of global emissions, are less dependent on fossil industries and have strong democratic norms, low corruption levels and high administrative capacity, but have politically powerful constituencies resisting ambitious climate policies, yielding uneven transition outcomes.

The remaining three clusters account for only 14% of emissions. The “Oil and Gas States” cluster (including Saudi Arabia, Iran, Algeria, and Venezuela), representing 6% of global emissions faces strong constraints on climate policy due to dependence on hydrocarbons for income and political stability. They are further characterized by low democratic governance and corruption control scores, limited climate awareness, and dominant resource nationalism narratives. The remaining two clusters—”Fragile States” and “Fractured Democracies” —together responsible for only 8% of global emissions, exhibit distinct features. Fragile states have weak state capacity and poor democratic norms, while fractured democracies, despite corruption challenges, show higher rates of climate legislation adoption.

Nascimento et al. [7] provided validating evidence for Lamb and Minx’s framework by examining 25 major emitters (80% of global emissions) selected from Lamb and Minx’s five clusters, finding that climate ambition and policy adoption aligned with constraint architectures: Oil and Gas States set unambitious targets; Coal-Dependent Development countries adopted conservative goals aligned with existing policies; Fragile States showed weak implementation; Fractured Democracies increased ambition without sufficient policy adoption; while Wealthy OECD nations demonstrated better ambition-implementation sequencing, but with significant variation.

While Lamb and Minx’s typology provides valuable insights into broad patterns of constraint, their clustering approach has limitations. For instance, Lamb & Minx [6] state that their focus is on climate mitigation but their inclusion of all countries—regardless of their actual contribution to global emissions—dilutes their findings. Many low-income nations have minimal carbon footprints; including these nations in a mitigation-focused analysis may obscure critical distinctions among higher-emitting countries.

Perhaps due to this, the 21 countries in their “Wealthy OECD” cluster exhibits striking internal variation despite the fact that they face “the lowest apparent levels of constraints” and therefore “ought to be leading in climate policy ambition”; this cluster contains “several declared climate ‘leaders’ such as the UK, Germany and Denmark” alongside relative laggards like the United States and Australia [6, p. 11]. Such divergence in performance within a supposedly homogeneous group suggests the presence of more nuanced constraint architectures than a five-cluster approach can capture.

Similarly, their ‘Coal Dependent’ cluster shows high internal diversity, containing 15 countries that—beyond coal use—are quite incongruous, such as Russia (a leading oil producer), China, and India (two countries at different levels of development and with divergent political institutions). The authors note that they stopped at five clusters because “beyond 5 we obtain only marginal differentiation between groups of wealthier northern states” [6, p. 7]. This choice, however, implies that potentially meaningful distinctions within and between country groups remain obscured. Indeed, the authors themselves recognize this limitation when they call for “deepening comparative political economy analysis to more nuanced and conditional constraints” on climate action [6, p. 11].

This suggests a clear need for a more granular analysis focused on developed economies, where distinct patterns of constraint may be operating beneath the surface of these wider classifications. Thus, our first step was to restrict our analysis to the countries covered by the Climate Change Performance Index (CCPI). The CCPI targets the 60 highest-emitting countries, which together account for more than 90% of global emissions. Starting with this pool ensures that the analysis remains centered on the countries most responsible for climate change. Within this group of high emitters, one region stands out as particularly consequential for global mitigation efforts: East Asia.

### East Asia’s energy transition challenge

**Fig 1:**
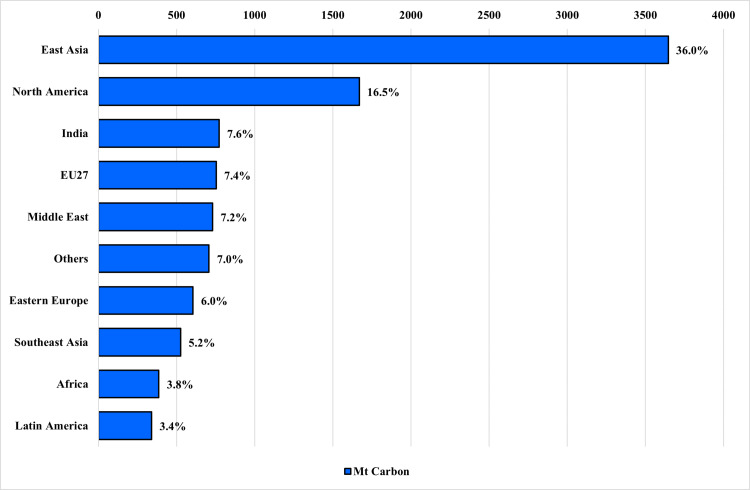
Regional CO_2_ emissions for 2022 in million tons of carbon per year [3].

The centrality of East Asia to global climate mitigation cannot be overstated. East Asia accounts for 36% of global CO_2_ emissions – more than twice that of North America (16.5%) and about five times that of the European Union (7.4%) (Fig 1). Clearly, the energy transition choices made by East Asian countries will largely determine the world’s ability to meet Paris Agreement targets.

Serving as the planet’s primary manufacturing hub, the region has traditionally fueled its robust economic growth with nuclear energy and fossil fuels, with coal alone representing between 30% and 63% of energy generation across the four major economies in the region [14]; (Table 1).

**Table 1:** 2022 Energy generation by fuel type as a percentage of total generation for East Asia.

Country	Oil	Natural Gas	Coal	Nuclear Energy	Hydroelectric	Renewables	Other	Total (TWh)
China	.14%	3.19%	62.56%	4.77%	15.23%	13.50%	.59%	8534.3
Japan	3.07%	31.98%	29.61%	6.00%	7.61%	12.78%	8.95%	1019.7
S Korea	1.17%	29.38%	35.26%	26.32%	.52%	6.70%	.68%	600.4
Taiwan	1.82%	37.23%	44.31%	9.56%	1.20%	4.16%	1.72%	290.9

Note: The ‘Total’ column represents the total electricity generation in Terawatt-hours for each country; other columns represent the percentage contribution of each fuel type to this total. Shaded areas correspond to the primary source of energy [14].

**Fig 2:**
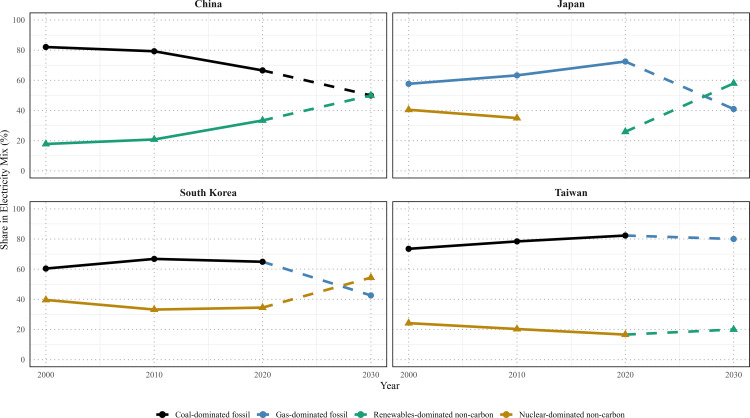
Sources of electrical power in East Asia, 2000–2030. The panels trace the evolution of the national electricity mix for China, Japan, South Korea, and Taiwan. Fossil-fuel shares (coal-, oil-, or gas-dominated, colored accordingly) and non-carbon shares (renewables- or nuclear-dominated) are plotted at four benchmark years: 2000, 2010, 2020, and the policy target year 2030. Solid lines connect historical observations (2000 → 2010 → 2020); dashed lines denote pledged values for 2030. Marker colors identify the dominant contributor within each aggregate at a given year (e.g., coal-dominated fossil share = black; gas-dominated = blue; renewables-dominated non-carbon = green; nuclear-dominated = dark yellow). Data compiled from national statistical yearbooks for Taiwan [22,23] and International Energy Agency databases for China, Japan, and Korea [24]. See details in S2 File.

East Asian governments have pledged to achieve renewable energy targets and enact supportive regulations (Fig 2, Table 2) but progress has been slow [12,15–18], hindered by infrastructure limitations, financial barriers, regulatory inconsistencies, and technological constraints [19–21]. As can be seen in Fig 2, China is the only East Asian nation currently on course to meet its energy transition targets.

**Table 2:** Overview of the net-zero target across a selected set of Asian countries.

Country	Net-zero target year	2030 target for cutting greenhouse gas emissions	Greenhouse gas emissions in 2022 (Percentage of world total)
China	2060	Reduce emissions per unit of GDP by over 65% from 2005 levels	29.2
Japan	2050	Reduce to 46% of 2013 levels	2.2
South Korea	2050	Reduce to 40% of 2018 levels	1.4
Taiwan	2050	Strive for ‘net zero’ emissions by 2050; Reduce 50% of carbon emissions below the 2005 level by 2050	0.75

Sources: International Energy Agency [25]; Taiwan’s 2022 share from Global Carbon Budget [3].

In Lamb’s framework, China is categorized within the ‘Coal development’ cluster, while Japan and South Korea (and presumably Taiwan, had it been included in the analysis) are situated in the OECD group. However, these three East Asian economies stand as distinct outliers within the broader ‘Wealthy OECD’ classification. We suggest that this divergence could be due to East Asia’s state-market regime, which prioritizes rapid export-led industrialization.

A long scholarly tradition has investigated how different political configurations shape late capitalist development [26–28]. Chalmers Johnson’s seminal account of Japanese postwar development extended this approach to East Asia, introducing the term ‘capitalist development state’ to describe how Japan’s powerful bureaucracy did not just regulate markets but actively shaped them. In his account, the Ministry of International Trade and Industry (MITI) led industrialization through administrative guidance, selective credit allocation, and strategic protectionism [8]. Amsden extended this approach to South Korea, demonstrating how the Korean state achieved rapid industrialization by deliberately “getting prices wrong”: using subsidies, credit rationing, export targets, and controlled exchange rates to lower costs for strategic industries while demanding performance standards in return [9]. Wade’s account of Taiwan’s development revealed how, even under different political conditions, a similar pattern emerged: an autonomous bureaucracy that “governed the market” through both leadership and followership, using state-owned enterprises, strategic credit, and selective liberalization to upgrade the industrial structure [10].

Across these accounts, East Asian developmental states—now including China [29]—share several defining features that distinguish them from Western models: (1) a cohesive, meritocratic bureaucracy with substantial autonomy from political interference; (2) pilot agencies that coordinate industrial transformation (MITI in Japan, Economic Planning Board in Korea, Council for Economic Planning and Development in Taiwan, and multiple coordinating bodies under China’s State Council); (3) state control over financial systems to direct credit toward strategic sectors; (4) “embedded autonomy” [30]—close consultation with business while maintaining state leadership; and (5) explicit prioritization of technological upgrading and export competitiveness over short-term profitability or consumer welfare. This institutional architecture created path dependencies that continue to shape climate and energy governance [31].

The legacy of developmental state characteristics is particularly evident in energy governance across East Asia, where the imperative of abundant, inexpensive energy for industrialization has created obstacles to the energy transition [32]. Recent scholarship has documented how these states systematically obstruct energy system decarbonization through a distinctive governance model that combines technocratic dominance with civil society exclusion. Across East Asia, energy policymaking remains captured by closed networks of bureaucrats and business elites, with minimal channels for public input despite growing environmental concern.

In Japan, a durable “METI–utility–industry” triangle dominates energy discourse, with pro-coal officials, utility federations (FEPCO), and business lobbies (Keidanren) effectively scripting policy outcomes before any public debate can occur [33,34]. This technocratic dominance is reinforced by regulatory cultures shaped by decades of top-down developmental governance, which have produced distinctive approaches to risk and public trust that persist post-Fukushima [35–37].

South Korea similarly manifests this pattern of civic exclusion: for example, despite high-level political commitments to net-zero, the target-setting process was marked by deliberately limited public consultation and engagement, underscoring what scholars identify as “the weakness of civic-participatory channels” [38]. Implementation remains incremental, facing resistance from entrenched bureaucratic institutions and industrial stakeholders who raise economic objections that effectively override public environmental concerns [38,39].

Taiwan’s energy transition also illustrates how developmental state structures marginalize civil society input. Dense state-business linkages—manifested through family-controlled conglomerates with government-appointed directors—have not only slowed renewable rollout but also blocked repeated attempts to introduce carbon-pricing instruments that enjoy public support [40]. The resulting combination of Taiwan’s developmental-era industrial coalition and popular anti-nuclear power sentiment has produced policy paralysis, with coal and LNG shares pushed above 80% despite public opposition [40–42].

China, with its more centralized party-state apparatus, exhibits this pattern of technocratic dominance over societal input even more completely. While the National Development and Reform Commission and National Energy Administration have made China the world’s dominant manufacturer of renewable technologies, coal still supplied 57% of primary energy in 2020, with expansion continuing under pressure from provincial governments and state-owned enterprises [43,44]. The Party justifies this contradiction through technocratic narratives of “Ecological Civilization” and “energy security” that frame decarbonization as a technological challenge rather than a matter requiring public participation [43].

This systematic exclusion of civil society is a defining characteristic of East Asian developmental states. Formal policies entrench fossil fuel dependence while informal practices favor incumbents and further insulate energy decisions from public pressure [33,34]. The result is what scholars describe as a preference for “incremental fixes over structural change” [34,39], where ambitious long-term targets coexist with minimal near-term action.

In East Asia, then, the developmental state has created a distinctive “architecture of constraint” where powerful state-business networks, technocratic policy monopolies, and systematic civic exclusion combine to block energy transitions despite growing public environmental concern. This configuration—high state capacity and public concern but weak civic channels—represents a combination of features that existing frameworks have not identified. If public opinion matters for climate policy in democracies, why do Japan, South Korea, and Taiwan lag so far behind despite their democratic institutions and environmentally concerned populations? And if East Asian states constitute such a distinctive regime type, why hasn’t this emerged in previous clustering analyses? We believe that one facet of what earlier analyses have lacked is a clearer set of indicators to reflect how ideas circulate within democracies to shape policy decisions.

### Democracy, civil society, and the flow of ideas in East Asia

The most influential accounts linking democracy to environmental policy outcomes derive from Western experiences in which public opinion is expected to influence policy directly through electoral competition and extra-parliamentary protest [4,5,45]. Recent large-N studies, however, suggest a more complex relationship between democratic institutions and emissions outcomes [46], with the effectiveness of electoral institutions being heavily mediated by development trajectories and fossil-fuel power blocs [47]. Such findings problematize the assumption that democracy automatically delivers deeper decarbonization.

East Asia lays this complexity bare. Japan, South Korea, and Taiwan excel by democratic measures, yet remain trapped in carbon legacies even as public concern in these countries grows. The reason, we argue, lies in decades of bureaucratic insulation and state–business collusion that have created policy venues in which electoral and protest signals are filtered out before they can influence policy-making. Climate governance remains the domain of unelected technocrats—epitomized by Japan’s METI—who coordinate with business interests through opaque ‘iron triangles’ linking ministries, heavy industries, and ruling parties [33,34]. Climate issues are framed as belonging to a ‘post-political’ technical realm, thus limiting democratic deliberation [34].

These barriers are reinforced by the relative weakness of grassroots political participation. Top-down state formation curtailed civil rights, leaving organized labor and consumer advocacy groups historically weak by comparative standards [9,10]. These factors ensured that even after democratization, climate policy remained captured by the original developmental coalition [48]. Channels for participation are often largely ceremonial: public comment periods and consultation sessions typically receive minimal response, referendum results are ignored, and civil society involvement remains ‘fragmented and piecemeal’ [35,49].

In short, East Asian countries, despite their differences, share a distinct developmental architecture of constraint, in which strong economic ministries and business-state alliances suppress civic-participatory channels. This configuration differs fundamentally from both autocratic fossil rentier states and pluralist Western democracies. New indicators may better capture this configuration of features.

Standard democracy indicators tell us who can vote, but not how environmental ideas travel from citizens to policy. We thus extend Lamb & Minx’s ‘ideas’ dimension by constructing new behavioral indicators using data from the 2020 International Social Survey Programme on the Environment [11]. First, we identify measures of environmental commitment, estimated through respondents’ self-reported willingness to sacrifice in the form of higher prices, taxes, or lower living standards. Next, we measure actual civic engagement through indicators of protest participation, environmental group membership, petition signing, and financial donations. Then, we develop a novel indicator for issue salience based on whether respondents rank environmental concerns among their top two national issues. Finally, we combine this salience measure with respondents’ self-reported party choices to gauge how effectively political parties reflect citizens’ environmental priorities. Our intent is to assess how well existing parties represent the environmental ideas and beliefs of their supporters in the political arena.

Employing these ISSP-derived indicators allows us to move beyond overly simplified conceptualizations of democracy, instead presenting democracy as a multi-dimensional construct composed of institutional channels through which ideas flow into the policy-making process. By measuring the civic and political pathways through which environmental ideas circulate—or fail to circulate—we aim to enhance the explanatory power of the original framework. Focusing on 28 high-emitting states responsible for approximately 70% of global CO₂ emissions further allows us to identify constraint architectures with greater precision.

## Materials and methods

### PCA-based clustering

We combine principal component analysis (PCA) and cluster analysis to uncover patterns of co-occurring national constraints and to classify countries into types with similar architecture of constraints [6]. This established technique in comparative political economy is useful for identifying institutional configurations where national variations are “not linearly distributed, but clustered by regime types” [50,51]. It is particularly suited for cases where multiple features form coherent, self-reinforcing systems. While most climate policy studies use regression analysis to isolate individual factors’ effects, East Asian developmental states exhibit tightly bundled features—state-business linkages, developmental ideology, and muted civic channels—each reinforcing the others, making it more appropriate to identify these configurations as integrated systems.

We therefore adopt Lamb & Minx’s exploratory workflow of (i) principal-component analysis to rotate correlated variables into orthogonal dimensions and (ii) clustering to identify countries that share similar scores along those latent axes. This approach highlights *configurations* of constraints or “architectures” and yields intuitive peer groups that inform policy prescriptions more directly than a table of regression coefficients. The trade-off is interpretive: clusters are descriptive rather than causal estimates, but that is appropriate for our research aim of mapping constraint typologies rather than estimating unit effects.

### Data

We compiled a dataset of countries with significant carbon footprints and robust measures of environmental discourse. We started with the Climate Change Performance Index (CCPI), which covers the 60 countries contributing most to climate change. For precise measurements of environmental ideas and institutional engagement, we used the 2020 International Social Survey Program on the Environment, which includes comprehensive data on environmental attitudes and representation across 28 countries [52].

We drew our dependent variables from the CCPI. The CCPI accounts for both policy ambition and real-world effectiveness, making it a robust and insightful tool for evaluating national mitigation efforts. While the inclusion of expert assessments has been criticized for introducing subjectivity [6], we consider this qualitative dimension a critical advantage. The CCPI evaluates policy commitment, implementation, and international engagement, aspects that purely quantitative measures—such as counting the number of climate laws—fail to reflect.

From the 2022 CCPI [12], we used four quantitative indicators, each assessing a different aspect of climate mitigation performance: *GHG Emissions Scor*e evaluates a country’s performance in reducing greenhouse gas emissions by assessing current per capita emissions, emission trends, compatibility with a well-below-2°C pathway, and 2030 targets; *Renewable Energy Score* measures the share of renewable energy in total energy use, its growth trend, and alignment with a well-below-2°C scenario; *Energy Use Score* tracks per capita energy consumption levels, trends, and how well they align with a sustainable transition; and *Climate Policy Score* evaluates a country’s climate policy effectiveness at both national and international levels based on expert assessments of policy ambition and implementation.

For our explanatory variables, we use variables representing configurations of interests, institutions, and ideas across different countries. To track interests, we use measures of countries’ dependence on fossil fuels and carbon-intensive energy. We include *fossil fuel subsidies* (explicit fossil fuel subsidies – total, as a percent of GDP [53]), which are financial expenditures supporting fossil energy, reflecting vested economic interests. In contrast to Lamb & Minx, who used this as a dependent variable, we classify subsidies as interests, since once implemented they become embedded in economic and political structures that favor continued fossil fuel use. With Lamb and Minx [6], we also include the *share of coal in electricity generation* [23], which captures supply-side coal dependency, but we do not include oil and gas rents [54] because they were collinear with the coal share.

For institutions, we start with updated versions of the indicators of governance quality and political context used by Lamb and Minx [6]. We used the Electoral Democracy Index (v2x_polyarchy) from the Varieties of Democracy (V-Dem) project, which measures the extent to which citizens can participate in free and fair elections, complemented by freedoms of expression, association, and universal suffrage [55,56]. We measure corruption with the 2022 WGI corruption index [57]. Next, to better capture the mechanisms through which public attitudes are channeled into policymaking, we added several measures drawn from the ISSP survey.

We further include a measure of *Environmental Representativeness of Parties*, using data from the ISSP 2020 [11], to reflect the extent to which existing political parties offer institutional channels for the expression of attitudes towards the environment. First, we identified respondents who selected the environment as either their first or second most important national concern (coded as ‘1’ in our binary “Salience” variable, with all others coded as ‘0’; see below for our discussion of salience). We then calculated the difference in the ISSP left-right party positioning (PartyL_R variable) between these environmentally concerned respondents and those who did not prioritize environmental issues. The absolute value of this difference serves as our indicator, with higher values indicating that people’s environmental attitudes are represented by the party that they select.

Finally, we add measures of *public environment-related participation* to better convey the routine institutionalized practices through which citizens may influence policy formation. Specifically, we include measures of whether respondents: (1) are members of an environmental organization; (2) have signed petitions about environmental issues; (3) have donated to environmental causes, or (4) have participated in environment-related protests [11].

While Lamb and Minx [6]) used the variables “social trust” and “climate awareness” from the World Value Survey to represent Ideas, we selected several variables measuring public opinion towards the environment from the ISSP 2020 [11]. First, we constructed a variable to measure the *salience* of the environment as an issue, which we have found provides a meaningful measure of environmental concern [58], perhaps because the items required making a choice that prioritizes different policy areas. We also attempt to gauge the depth of respondents’ beliefs through measures of willingness to sacrifice, in terms of willingness to pay higher prices and taxes, and willingness to accept reduced living standards.

All variables are shown in Table 3; further details are provided in the supplementary data file, S1 File.

**Table 3:** Study variables.

Theoretical Variable	Empirical Variable	Description	Year
**Dependent Variables**			
GhG Emissions	GhGs_CCPI1	CCPI GhG emissions score.	2021
Renewable Energy Trends	Renewables_CCPI2	CCPI measure combining renewable share and growth trend.	2021
CO2 Intensity	EnergyUse_CCPI3	CCPI measure tracking energy consumption levels and trends.	2021
Climate Policies	ClimatePolicy_CCPI4	CCPI evaluation of climate policy effectiveness.	2021
**Interests**			
Coal in Electricity Mix	CoalShare	Percentage of Coal in Total Electricity Mix	2022
Fossil Fuel Subsidies	FossilSubsidies	Explicit Fossil Fuel Subsidies – Total, Percent of GDP	2022
**Institutions**			
Electoral Democracy Index	Democracy	The v2x_polyarchy variable from the V Dem project.	2022
Control of Corruption	Corruption	WGI Control of Corruption variable.	2023
Environmental Representativeness of Parties	Representativeness	Extent to which political parties represent environmental views.	2019-2022
Membership in Environmental Organizations	Member	Members of environmental group as percent of country population	2019-2022
Signed Petitions Related to Environmental Issues	Petition	Percent of population that signed petition for environmental cause	2019-2022
Made Donations to Environmental Organizations	Donation	Percent of country population that donated for environmental cause	2019-2022
Participated in Protests on Environmental Issues	Protest	Percent of country population that attended a protest for environmental cause	2019-2022
**Ideas**			
Salience	Salience	Percent in country population selecting environment as first or second most important issue	2019-2022
Willingness to Sacrifice (Prices)	Prices	Average willingness to pay higher prices	2019-2022
Willingness to Sacrifice (Taxes)	Taxes	Average willingness to pay higher taxes	2019-2022
Willingness to Sacrifice (Standard of Living)	LivingStandard	Average willingness to reduce standard of living	2019-2022

### Analysis

Before analysis, all data series were reviewed for completeness and consistency. As Iceland was not included in the CCPI and Taiwan is not included in the SDG datasets, their values on the corresponding variables were estimated using two methods. First, we imputed the missing values, using k-Nearest Neighbors (k = 3) imputation with the VIM package in R, which estimates missing values based on similar data points. Second, we used external data for all of Iceland’s missing values (Greenhouse emissions per capita, renewable share, CO_2_ intensity, and climate policies from the International Energy Agency [23]) and for two of Taiwan’s four missing values (greenhouse emissions per capita and renewable share, from Taiwan’s Ministry of Economic Affairs [23]); and used the imputed values for the remaining two as no external sources were available. We then compared results across these two methods and found no meaningful differences. We thus used the k-Nearest Neighbors imputations for all missing values in our subsequent analyses.

We scaled all quantitative variables to standardize units and variance (mean of 0, standard deviation of 1) prior to analysis to ensure that no individual variables dominated the analysis due to scale differences. After preparing the dataset, we implemented a three-step quantitative analysis to extract key dimensions and group countries by their constraint profiles:

**Spearman Correlation Analysis:** A Spearman correlation analysis among all variables was conducted. The correlation matrix was visually inspected using a clustered correlation heatmap to confirm expected associations and to identify any extremely collinear pairs or groupings among the indicators. The dataset was found to provide a multifaceted but not redundant representation of the Three I’s in each country.

**Principal Component Analysis (PCA):** We performed a principal component analysis on the scaled data to identify underlying composite dimensions of national climate policy constraints. By using PCA, we mitigate multicollinearity among the variables and create uncorrelated factors that serve as a cleaner basis for clustering. We also avoid overweighting any single indicator, since each principal component is a weighted mix of the original variables. The correlation circle analysis obtained informed the subsequent PCA by confirming the contributions of the selected variables. We used the Prcomp algorithm in R with centering and unit variance scaling so that each variable contributes proportionately. The PCA results were examined via eigenvalues and explained variance. We retained the top components that collectively accounted for at least 85% of the variance in the dataset to ensure that most of the information is preserved while filtering out noise.

(We initially included fossil fuel rents as a percentage of GDP using World Bank data [54] and an ISSP 2020 measure of trust [11]. However, in our preliminary analyses, the Spearman correlation analysis showed high multicollinearity between trust and democracy, and between fossil fuel rent and coal share. The PCA further showed these variables as superimposed. We therefore removed both variables).

**Cluster Analysis:** Multiple methods were used to determine the optimal number of clusters: the elbow method (within-cluster sum of squares), silhouette analysis, gap statistic, and the NbClust package’s majority rule approach across multiple indices. Based on these diagnostics, we implemented K-means clustering with k = 6, using 25 random starts to avoid local optima. Cluster validity was assessed using silhouette width, which measures how well observations fit within their assigned clusters. We compared K-means results with hierarchical clustering using Ward’s method to verify the robustness of the cluster solution. Cluster profiles were generated by calculating mean values of original variables for each cluster to facilitate interpretation of the resulting country groupings.

Additionally, we employ two distinct measures of climate performance: the Climate Change Performance Index [18] as our primary measure and Sustainable Development Goals (SDG) 7 and 13 indicators [59] as a robustness check. In contrast to the CCPI variables, the SDG indicators provide more narrowly focused, technically specific metrics without explicit policy components: SDG 7 indicators measure CO₂ emissions per electricity output and renewable energy share in final consumption, while SDG 13 indicators capture fossil fuel and cement production emissions and emissions embodied in imports. The key distinctions between these measurement approaches are threefold: the CCPI incorporates forward-looking assessments of alignment with climate targets, includes explicit policy evaluation dimensions, and employs expert judgment alongside quantitative metrics. The SDG indicators, while more limited in scope, offer precise, comparable measurements of emissions across different economic sectors and consumption patterns. See S2 File for the methods and results of this analysis.

All computations were conducted in R [60].

## Results

To explore the internal structure of our indicators prior to dimensionality reduction, we computed pairwise Spearman correlations among 17 variables spanning climate performance (CCPI dimensions), interest-based constraints (coal share, fossil fuel subsidies), institutional quality (democracy, corruption, civic engagement including membership, petitions, donations, protests), and idea-level support (salience and willingness-to-sacrifice: increase prices and taxes/cut standard of living).

**Fig 3:**
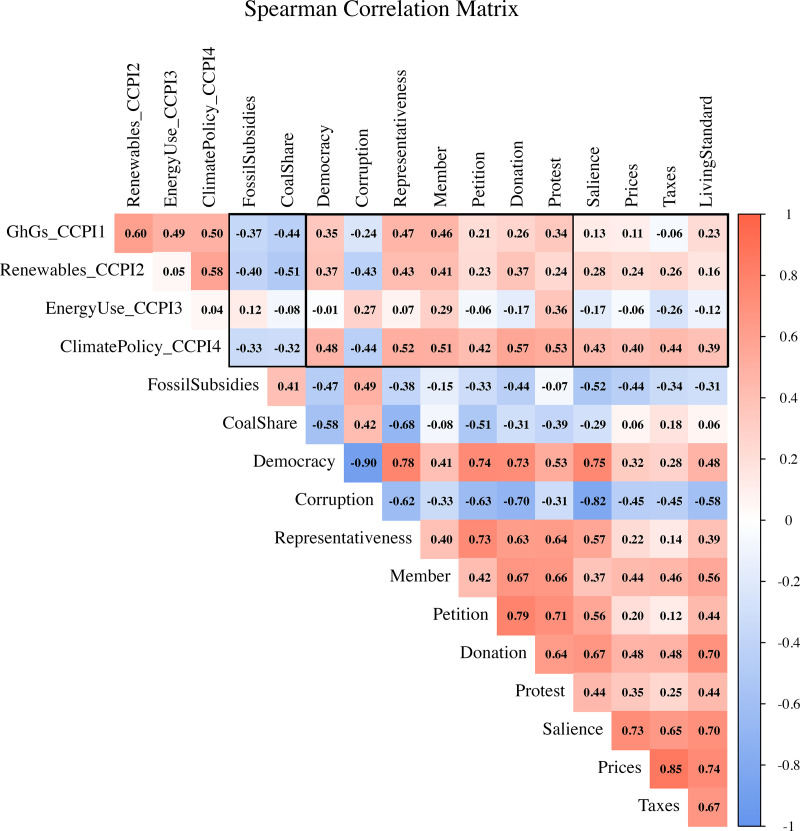
Spearman correlation matrix. The black borders indicate variable sets measuring, from left to right, interests, institutions, and ideas.

As shown in Fig 3, correlations between institutional quality and climate performance are generally weak to moderate (ρ = 0.01–0.57), with the strongest links observed for the climate policy and renewable energy sub-indices. None of the institutional or ideational variables correlate strongly (ρ > 0.60) with any CCPI dimension. Even the most aligned outcome—climate policy—is only moderately associated (ρ = 0.40–0.57) with variables such as democracy, corruption control, environmental salience, and willingness-to-sacrifice. These patterns suggest that climate performance is not driven by any single institution or attitude factor, but rather emerges from the interaction of multiple, overlapping constraints.

Idea-level indicators—salience and willingness to accept higher prices, taxes, or lower living standards—are consistently interrelated, suggesting a latent attitude dimension of environmental commitment. However, their associations with climate performance measures remain modest.

Fossil fuel interest proxies, particularly coal share and fossil subsidies, correlate negatively with both institutional quality and climate policy performance. Civic engagement variables display a mixed pattern: while membership, petitioning, and donation show moderate correlations, protest participation exhibits poorer, more inconsistent associations, possibly reflecting a distinct dynamic.

A small number of very strong correlations (|ρ| > 0.70) emerge indicating tightly coupled institutional or behavioral dimensions: most notably the negative association between democracy and corruption (ρ = –0.90), as well as high positive correlations between willingness to pay higher taxes and prices (ρ = 0.85), petitioning and group membership (ρ = 0.79), and petitioning and donation (ρ = 0.71).

These descriptive results provide the basis for our subsequent dimensionality reduction.

**Fig 4:**
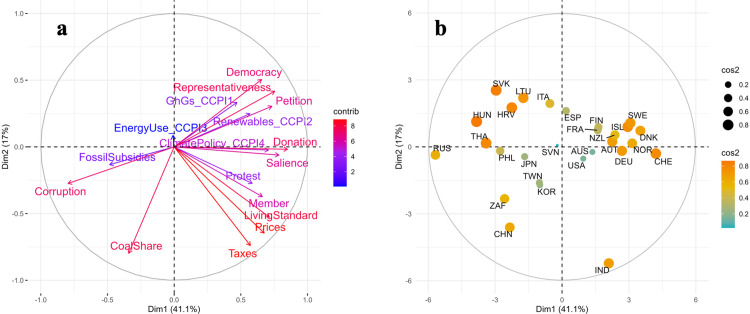
PCA of climate policy factors: Variable loadings and country positions. **a. Correlation Circle (Left):** Variable loadings on Principal Components. Arrows indicate correlations between the original variables and the principal components. The unit circle (radius = 1) represents perfect correlation. Color intensity indicates each variable’s contribution to defining these components. **b. Countries Plot (Right):** Country positions on Principal Components. Values represent standard deviations from the mean (0,0). Countries with similar patterns cluster together. Both color (red-orange to blue-green) and point size indicate quality of representation (cos²) in this two-dimensional space, with larger, red-orange points showing better represented countries and smaller, blue-green points showing countries that additional dimensions may better characterize.

Fig 4 presents the results of our principal component analysis (PCA), combining the variable loadings (panel a) and country positions (panel b) on the first two principal components.

The correlation circle (Fig 4a) illustrates the relationships between the variables and the first two components. PC1 is characterized by high positive loadings (≥ |0.30|) for a range of institution and idea variables, including democracy, salience, petition, donation, and climate policy, while displaying high negative loadings for corruption and fossil subsidies. PC1 thus represents a contrast between enabling institutional and ideological conditions for climate action and entrenched interest-based constraints. PC2 opposes coal share (loading = –0.47) with willingness-to-sacrifice items such as taxes, prices, and living standards (loadings between –0.31 and –0.43), suggesting a second axis defined by public support for cost-bearing mitigation measures versus fossil dependence. Several civic engagement indicators (e.g., protest, membership) and CCPI performance components (e.g., renewables, energy use) show weaker or more diffuse loadings, indicating limited contribution to the first two axes and greater relevance in later components.

Table 4 summarizes the loadings of each climate policy determinant across the first six components. The first principal component (PC1) accounts for 41% of the total variance, followed by PC2 with 17%; together, they explain 58% of the variation in the dataset. The first six components, taken together, account for 88%, providing an appropriate basis for dimensionality reduction.

**Table 4:** Principal component loadings for climate policy determinants.

	PC1	PC2	PC3	PC4	PC5	PC6
Standard deviation (SD)	2.64	1.7	1.46	1.18	0.99	0.75
Proportion of variance	0.41	0.17	0.13	0.08	0.06	0.03
Cumulative variance	0.41	0.58	0.71	0.79	0.85	0.88
**Dependent Variables**						
GhGs_CCPI1	0.18	0.20	**0.46**	−0.16	0.14	−0.25
Renewables_CCPI2	0.22	0.15	0.12	**−0.39**	**0.52**	−0.15
EnergyUse_CCPI3	0.00	0.05	**0.57**	0.03	**−0.45**	**−0.32**
ClimatePolicy_CCPI4	0.27	−0.01	0.17	**−0.32**	0.10	**0.41**
**Interests**						
FossilSubsidies	−0.18	−0.08	0.05	**0.54**	**0.45**	−0.23
CoalShare	−0.13	**−0.47**	0.03	−0.11	−0.11	0.22
**Institutions**						
Democracy	0.25	0.30	−0.13	0.00	**−0.38**	−0.04
Corruption	**−0.30**	−0.16	**0.31**	0.13	0.13	0.04
Representativeness	0.29	0.25	0.04	0.15	0.11	0.06
Member	0.25	−0.22	**0.31**	0.12	0.14	0.10
Petition	0.28	0.18	−0.06	**0.39**	−0.09	**0.33**
Donation	**0.32**	−0.01	−0.07	0.24	0.22	0.16
Protest	0.22	−0.16	**0.34**	0.29	−0.17	0.21
**Ideas**						
Salience	**0.30**	−0.04	−0.26	0.06	−0.05	**−0.46**
Prices	0.26	**−0.38**	−0.09	−0.07	−0.04	−0.24
Taxes	0.22	**−0.43**	−0.07	−0.20	−0.02	0.04
LivingStandard	0.27	**−0.31**	−0.07	0.16	−0.02	−0.27

Note: Bold values indicate loadings with absolute values ≥ 0.30. These higher loadings highlight which variables contribute most significantly to each principal component.

The country projection (Fig 4b) shows how countries position themselves along these two dimensions, with each point’s size and color reflecting its cos² score—i.e., the quality of representation on the PC1–PC2 plane. Countries that appear close together share similar profiles across the original variables, while those further from the origin and more brightly colored are better represented by these two components. For instance, Sweden is strongly represented on the pro-climate, institutionally supportive side of PC1, whereas Russia exemplifies countries with high fossil interests and weak institutional quality. Countries with low cos² scores may be better characterized by higher-order components, which are addressed in the following analysis.

Beyond the first two dimensions, the third to sixth principal components (PCs 3–6) account for 30.9% of the total variance. As shown in Table 4, PC3 is primarily defined by strong positive loadings on GHG emissions (0.46), energy use (0.57), and protest participation (0.34), alongside a moderate negative loading for salience (−0.26), suggesting a contrast between material consumption and attitude concern. PC4 features high positive contributions from fossil subsidies (0.54) and petition (0.39), and negative loadings on climate policy (−0.32) and renewables (−0.39), suggesting a dimension where environmental activism may be a response to weak climate policies. PC5 captures variation linked to renewables (0.52) and energy use (−0.45) and loads positively on fossil subsidies (0.45) and negatively on democracy (−0.38), pointing to a mixed axis of energy structure and political environment. Finally, PC6 highlights additional variance in climate policy (0.41), petition (0.33), and salience (−0.46), suggesting a dimension where policy ambition and public concern diverge. Japan, South Korea, and Taiwan, for instance, are better represented on PCs 3 and 4, reflecting high energy use, modest civic engagement, and limited policy ambition. In contrast, New Zealand and Lithuania stand out on PC5 and PC6, due to their stronger climate policy scores and differentiated civic profiles.

The PCA results provide a reduced-dimensionality representation of the complex relationships among institutional, interest-based, and ideational variables. By extracting six principal components that together explain 88% of the total variance, we retain the most informative and uncorrelated dimensions of variation while minimizing redundancy. This approach lets us identify countries that face similar overall configurations of climate policy constraints, grouping together nations with comparable patterns across all dimensions.

**Fig 5:**
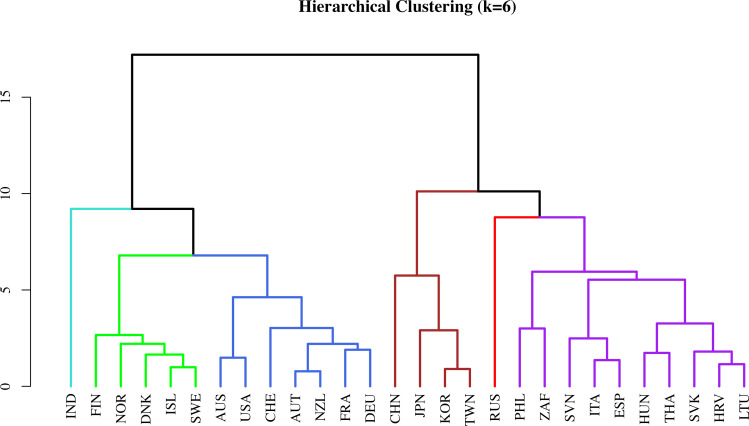
Dendrogram showing the six clusters of countries.

We compared k-means and hierarchical clustering solutions across multiple values of k, using silhouette width and consistency as criteria. At k = 6, both methods showed the highest level of convergence, with the Rand Index and Adjusted Rand Index equal to 1, indicating perfect agreement in-country assignments. Importantly, k = 6 also offered the most interpretable and policy-relevant classification, distinguishing between high-capacity and structurally constrained climate actors without excessive fragmentation. Fig 5 displays the hierarchical clustering dendrogram based on the six principal components.

**Fig 6:**
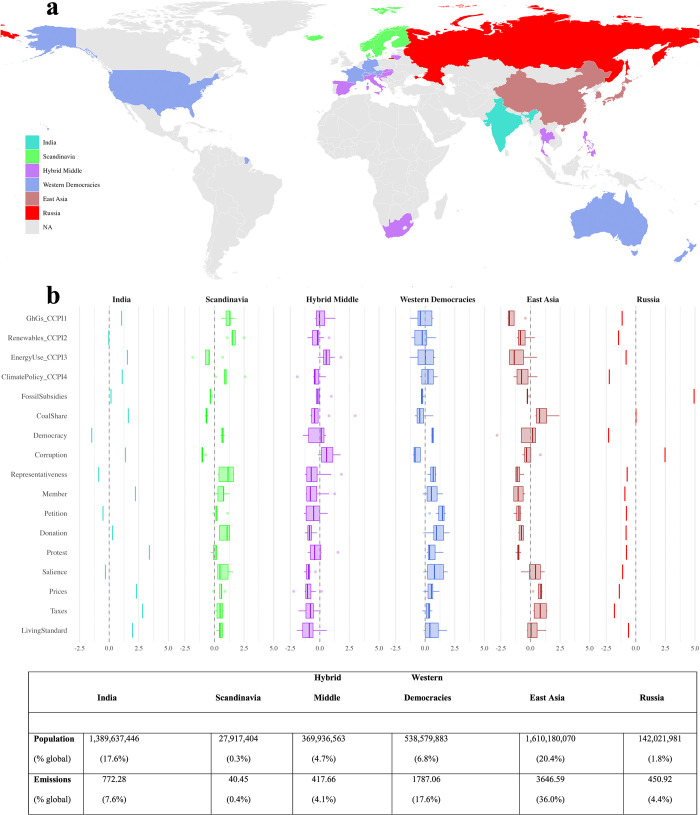
Clusters of constraints across countries. Note: Total 2022 population and emissions data in million tons carbon per year for each cluster are shown, but are not used as variables in the cluster analysis. Sources: Population data is from World Bank [61]; emissions data from Global Carbon Budget [3]. This figure shows the spatial distribution of constraint clusters across countries. The base map, showing national boundaries, is sourced from Natural Earth (public domain; https://www.naturalearthdata.com) and the figure was generated by the authors using the rnaturalearth R package.

Fig 6a maps the geographic distribution of the six clusters, and Fig 6b presents their average profiles across 17 climate-related indicators. In the dendrogram, the ordering from left to right is not interpretively meaningful. Therefore, in Fig 6b, we order the clusters in terms of their overall CCPI score, with the highest performer (India) on the left and the lowest (Russia) on the right.

In Fig 5, India forms a unique cluster characterized by moderate institutional capacity and significant coal dependence. Yet, it also exhibits surprisingly strong indicators of willingness to sacrifice and civic engagement, especially in terms of membership and protest activity. Its overall emissions remain very low, a reflection of India’s level of economic development to date. How this constraint architecture evolves as incomes rise—in a country that accounts for nearly a fifth of the global population—will be critical for global mitigation efforts.

The Scandinavia cluster (Denmark, Finland, Iceland, Norway, and Sweden) exhibits high scores across all climate performance indicators, particularly in climate policy ambition and renewable energy deployment. These countries also feature strong democratic institutions, low levels of corruption, minimal entrenchment of fossil fuel interests, high environmental salience, and moderate to high levels of civic engagement. While exemplary in their transition efforts, these countries together account for just 0.4% of global emissions (Fig 6b) – too small to significantly influence global outcomes.

The Hybrid Middle cluster groups ten countries across Europe, Asia, and Africa (Lithuania, Hungary, Croatia, Slovenia, Slovakia, Italy, Spain, the Philippines, Thailand, and South Africa), characterized by mixed climate performance, along with moderate levels across interests, institutions, and ideas. Salience, willingness to sacrifice, and civic engagement are comparatively low. The diversity within this cluster suggests that factors beyond our framework may further differentiate their climate trajectories.

The Western Democracies cluster (Australia, Austria, France, Germany, New Zealand, Switzerland, USA) shows low to moderate fossil fuel interests, good institutional quality alongside strong salience and civic engagement, with an overall profile similar to that of the Scandinavian cluster. Yet, in contrast to the Scandinavian cluster, climate performance remains surprisingly low to moderate.

The East Asia cluster (China, Japan, South Korea, and Taiwan) is marked by high coal dependence and moderate institutional capacity. While public concern is high—reflected in strong salience and willingness to sacrifice—civic engagement and political representation remain low. Climate performance is strikingly poor, a disturbing reality given the size of the region’s carbon footprint. Notably, our results show that Japan, South Korea, and Taiwan cluster more tightly together, while China’s position likely reflects its unique combination of developmental state features with authoritarian governance (Fig 5).

Finally, Russia forms a standalone cluster characterized by extremely high fossil subsidies and reliance on coal, low institutional quality, and minimal public or civic support for climate action. Unsurprisingly, Russia has the weakest climate policy scores in the dataset.

Finally, our robustness analysis using SDG indicators reproduced all six of the above macro-clusters and their defining characteristics, confirming that our typology is not an artefact of indicator choice or cluster granularity (see S2 File for full methods and results).

## Discussion

Building upon Lamb and Minx’s concept of “architectures of constraint,” which examines how interlocking interests, institutions, and ideas jointly condition national variation in climate policy ambition [6]—this study introduces a higher-resolution lens to this analytical framework. While Lamb and Minx conducted a global analysis of 99 countries covering 92% of global emissions and identified five broad clusters, our dataset includes 28 countries that nonetheless account for approximately 70% of global carbon emissions. Our PCA/cluster analysis approach identified a new six-cluster typology of high-emitting countries. This six-cluster solution refines the five-category framework of Lamb and Minx [6] in two important ways. First, by focusing on high-emitting countries, we provide a more detailed analysis of the most important countries in the energy transition. Second, by incorporating new variables on civic engagement and political representation of environmental issues (derived from ISSP), we capture the idea-to-policy transmission mechanisms that are often missing in discussions of the role of democratic politics in the energy transition. The results identify a distinct cluster comprising East Asian developmental states (China, Japan, South Korea, Taiwan), which did not emerge in previous analyses despite accounting for 36% of global carbon emissions.

### The East Asian developmental state cluster: A distinct architecture

China, Japan, South Korea, and Taiwan exemplify a distinctive “developmental state” model whose institutional architecture is associated with underperformance in the energy transition. These countries possess strong bureaucracies and advanced technological capacities, yet their governance remains shaped by a postwar legacy of state-led industrialization that prioritized economic growth at all costs. This paradox of high capacity but lagging climate action defines the East Asian developmental state cluster [48,49].

Three core institutional features distinguish this model and help contextualize why environmental concern fails to translate into policy action. First, a technocratic bureaucracy operates with considerable insulation from external scrutiny. Policy agendas are largely set by elite economic agencies that prioritize industrial objectives with minimal public oversight. Epitomized by Japan’s METI, these agencies continue to script energy policy through closed “iron triangles” that link ministries, utilities, and business lobbies [33,34]. Second, tight state-business linkages facilitate coordination with incumbent sectors while marginalizing alternative voices. Ministries retain close ties with fossil-intensive industries and consistently prefer voluntary, investment-driven approaches over regulatory constraints that might threaten established interests [40,41,62,63]. Third, systematic exclusion of civil society from policymaking ensures that environmental advocacy remains politically marginalized. Decades of top-down state formation curtailed grassroots political participation, leaving organized environmental groups historically weak even after democratization [48,49,64].

These institutional features produce a distinctive pattern of climate governance where symbolic ambition substitutes for structural transformation. Governments embrace green growth rhetoric and long-term net-zero targets, but these remain decoupled from binding near-term goals or politically difficult decisions, such as coal phase-outs—a hybrid model of “developmental environmentalism” that promotes green technology as a new industrial frontier while leaving legacy emissions largely untouched [41].

### Making democracy effective: The role of civic engagement and party representation

It is typically assumed that democracies deliver stronger climate policy outcomes because public opinion in favor of environmental protection can influence policymaking. In this view, democratic institutions facilitate the translation of citizens’ preferences into government action, leading to ambitious climate commitments. Empirical studies generally support these claims. Neumayer [4], for example, finds that democracies ratify more international environmental agreements than autocracies, reflecting a higher level of “environmental commitment.” Other cross-national research shows that public concern about climate change is a significant predictor of policy stringency in democratic settings [65,66]. At both national and subnational levels, there is evidence that democratic governments respond to pro-environment public opinion: U.S. states with greater environmental movement activity and public support passed more environmental legislation [67], while variation in public demand for climate action explains differences in adopted climate policies among democracies [68]. Such studies assume that the mechanisms of democracy ensure that as citizens become more environmentally conscious, their governments will enact stronger climate policies [69,70].

Our findings complicate this optimistic picture by revealing that, in democracies such as Japan, South Korea, and Taiwan, pro-environmental public attitudes do not translate effectively into climate policy outcomes. What is missing in the East Asian developmental state model is strong civic-participatory channels for environmental advocacy and political representation in the form of party platforms that meaningfully represent pro-environmental positions. While public concern matches Western levels, our analysis reveals striking gaps between environmental concern and civic mobilization in East Asia. On average, only about 2% of respondents in Japan, South Korea, and Taiwan report membership in environmental organizations, compared to roughly 9% in Western democracies. Similarly, engagement in environmental petitions (8–10% vs ~ 33%), donations to environmental causes (~7% vs ~ 24%), and protest participation (~1% vs ~ 8%) is strikingly low—approaching levels observed in authoritarian Russia.

Perhaps most revealing is our Environmental Representativeness index, which measures whether political parties offer meaningful channels for expressing environmental attitudes. East Asian democracies score near zero: Japan (0.01), Taiwan (0.06), and South Korea (0.19)—comparable to Russia (0.14). By contrast, Western democracies like France (0.67) and Germany (0.65) exhibit strong partisan polarization on environmental issues, indicating that voters who prioritize the environment gravitate toward distinctly different parties than those who do not. In East Asia, environmental concern remains weakly represented in party competition, reflecting the broader marginalization of civic input in developmental state governance.

These patterns indicate that public support for climate action is not absent in East Asia; rather, it is structurally muffled by an architecture in which policymaking remains elite-driven, as has been extensively documented in the existing literature [21,33,34,38,41,64]. This developmental state paradox suggests that the specific channels through which citizens influence policy—not merely the formal presence of democratic institutions—may shape climate outcomes, challenging assumptions about democracy as a monolithic category in climate governance.

Our findings caution against lumping all democracies together in climate governance research. The cluster analysis of 28 countries clearly identifies divergent pathways among democratic systems, with three distinct subsets of democracies emerging, in addition to the single case of India. One cluster comprises the high-performing Scandinavian countries, which pair strong democratic institutions with deeply embedded pro-climate norms—a “virtuous alignment” of state capacity, public engagement, and political will. A second grouping covers other Western democracies, characterized by robust civil societies but also by partisan polarization and policy oscillation as governments flip between left- and right-leaning agendas (e.g., Australia, Germany, the U.S.). The East Asian developmental states form a third, quite different cluster: three of the four countries (Japan, South Korea, and Taiwan) are wealthy democracies with, however, top-down governance structures and muted societal input, characteristics associated with slower climate policy progress [41,64]. These distinct democratic pathways underscore that climate governance strategies cannot be one-size-fits-all. Effective climate action requires tailored approaches for each type of democracy.

### Policy implications and the paradox of reform

At the same time, our analysis reveals a fundamental challenge: the constraint architectures we identify—weak civic channels, limited environmental representation, and entrenched state–business networks—suggest that conventional climate policy recommendations premised on responsive democratic feedback are unlikely to gain traction. The very reforms that might accelerate climate action (strengthening civil society, enhancing participation, reducing capture) are precisely those that existing institutions resist. The policy avenues discussed below should therefore be understood not as prescriptions arising from public preference signals, but as pathways that operate around these constraint configurations—a practical reality that international climate governance must confront.

Nevertheless, it is important to seek pathways that function within these constraints. Adopting a complex adaptive systems perspective [71,72] offers a crucial insight here: while developmental state architectures are deeply entrenched, they are not static. As dynamic systems, they interact with their environments in ways that can produce non-linear changes. This reinforces the practical importance of crisis-driven windows of opportunity, such as the Fukushima disaster or acute episodes of air pollution, which can temporarily destabilize established networks and create openings for reform [36]. Consequently, climate governance must be understood as operating within rapidly shifting policy windows [73], where geopolitical tensions, energy crises, or technological breakthroughs can quickly alter constraint architectures. For policymakers, the implication is clear: rather than waiting for gradual institutional evolution, they must prepare concrete proposals in advance to leverage these moments of systemic disruption.

Given the top-down nature of East Asian governance, climate initiatives framed as technological leadership or economic competitiveness may gain more traction than those emphasizing democratic participation. China’s massive renewable investments exemplify this industrial-policy approach [74,75], suggesting that reframing climate action as national competitiveness can bypass political bottlenecks. However, China’s deep structural contradictions remain unresolved: rapid renewable expansion has occurred alongside persistent coal reliance and significant gaps between central targets and provincial implementation [76,77]. Importantly, this quantitative progress has not opened channels for civic influence or improved translation of public environmental concern into policy. Thus, while the ‘competitiveness’ frame may effectively mobilize investment, China’s experience suggests that without broader institutional checks, high-tech green growth may overlay, rather than replace, the carbon-intensive legacy economy.

A third potential pathway is through external pressure in the form of trade linkages, such as EU carbon border adjustments, and technology partnerships to create incentives that bypass domestic political bottlenecks [78]. Additionally, subnational governments may have more flexibility to experiment with participatory approaches that could eventually scale up [79]. Finally, East Asian countries could establish new climate-focused institutions that operate alongside traditional bureaucratic structures, such as Taiwan’s renewable energy offices or South Korea’s Green New Deal bodies, gradually shifting the balance of influence.

Crucially, we must recognize that East Asia’s climate transition will follow a fundamentally different path than Western models—one likely to rely more on state capacity and technological deployment than on civic mobilization [80]. The key insight for international climate governance is that strategies must be tailored to these institutional realities rather than assuming universal applicability of participatory, market-based approaches. This means rethinking how global climate frameworks engage with different political economies and recognizing that effective climate action may emerge through diverse institutional pathways, even those that challenge conventional assumptions about the relationship between democracy and environmental policy.

### Limitations and future research

Our analysis has several limitations. First, regarding case selection, we focus on 28 high-emitting countries with available ISSP data, excluding major emitters like Iran, Indonesia, Canada, Mexico, and Brazil. The ISSP survey questions do not align perfectly with our climate focus, and some variables serve as proxies for complex concepts. Consequently, while some clusters (East Asian, Scandinavian) appear institutionally coherent, others (particularly Hybrid Middle) lack clear shared trajectories, possibly reflecting variable selection rather than structural similarity.

Second, while our clustering approach identifies countries with similar constraint architectures, we acknowledge that significant diversity exists within these groups. The countries in our East Asian cluster, for instance, exhibit distinct differences in their specific energy mixes, civil society landscapes, and political regime types. Future research should therefore explore how these internal variations interact with the broader framework of the developmental state to shape distinct policy outcomes.

Third, our cross-sectional design captures constraint architectures at a single point in time (2019–2022), yet as the literature on complex adaptive systems suggests, these institutional configurations are not static [71,73]. East Asian political systems are undergoing internal shifts: China exhibits tension between technocratic professionalism and centralized control [81,82], alongside elements of “responsive authoritarianism” [83,84], while Japan’s bureaucratic system has experienced dramatic transformations in the balance between political leadership and bureaucratic autonomy [85,86]. However, CCPI (Climate Change Performance Index) trajectories from 2010 to 2026 reveal no marked changes in East Asian country rankings (except for slight improvement in China’s ranking, likely due to renewable additions) suggesting that such changes have not necessarily disrupted the lack of connection between grassroots environmental preferences and institutional policymaking. Our typology should therefore be understood as an analytical snapshot of durable, yet evolving, constraint configurations, rather than immutable regime types.

Fourth, we recognize that institutional and attitudinal variables can only provide a partial explanation for climate outcomes. A comprehensive understanding requires incorporating structural economic factors—such as trade patterns, industrial specialization, and global production networks [80]—which we could not fully account for here. Furthermore, given our cross-sectional design, our findings should be interpreted as illuminating the political constraints that interact with these broader drivers, rather than establishing strong causal inferences.

Ultimately, the most urgent questions for future research are practical ones: How do you actually achieve climate action within a technocratic East Asian developmental state versus a polarized Western democracy? While current climate governance often assumes the universal applicability of participatory, market-based models, each constraint architecture likely requires fundamentally different strategies. Future work might investigate how institutional legacies can be leveraged rather than circumvented. Such research might ask, for example, if East Asia’s technocratic capacity can be redirected through industrial policy frames, or how crisis moments can be best exploited to disrupt established constraints. Given that East Asian developmental states control over one-third of global emissions, understanding how to unlock climate action within these distinct institutional realities is critical.

## Conclusion

Our study deepens the critical conversation surrounding climate governance by refining and expanding upon the “architectures of constraint” framework. By identifying distinct democratic clusters, particularly the unique institutional configuration of East Asian developmental states, we shed further light on why some nations lead decisively on climate action, while others lag behind. The East Asian cluster, characterized by strong bureaucratic control, limited civic mobilization, and minimal political representation of environmental concerns, emerges as a crucial determinant of global climate outcomes. Given that this group alone largely determines global emissions trajectories, addressing the constraints within East Asian democracies is an existential imperative. Future research and climate policy must engage with this institutional reality. Ultimately, the fate of the global energy transition—and indeed, the planet itself—may hinge on whether and how these nations pivot towards effective climate action.

## Supporting information

S1 File Country-level dataset that underlies all analyses in the manuscript. (XLSX)

S2 File Supplementary tables and figures. (DOCX)
