# Supplementary material for: Technocrats vs. tipping points: How East Asian governance shapes global emissions
Source: PLoS One. 2026 Jan 20;21(1):e0339968. doi: 10.1371/journal.pone.0339968 (PMC12818633; doi:10.1371/journal.pone.0339968)
Supplement: S2 File — (DOCX) [file pone.0339968.s002.docx]

**S2 Appendix**

**Table of Contents**

1. **Electricity Mix for China, Japan, South Korea and Taiwan**
2. **Robustness Check**

**1. Electricity Mix for China, Japan, South Korea and Taiwan**

**S2 Table 1.** Details of the sources in the electricity mix of China, Japan, South Korea and Taiwan

for 2000, 2010, 2020 and pledges for 2030.

| **Year** | **Country** | **Coal**  **(%)** | **Oil**  **(%)** | **Natural Gas (%)** | **Nuclear (%)** | **Renewable (%)** | **Other**  **(%)** |
| --- | --- | --- | --- | --- | --- | --- | --- |
| 2000 | China | 78.2 | 3.5 | 0.4 | 1.2 | 16.6 | 0 |
|  | Japan | 21.4 | 12.5 | 23.8 | 30.2 | 10.3 | 1.9 |
|  | S. Korea | 38.2 | 12 | 10.2 | 37.7 | 1.9 | 0 |
|  | Taiwan | 47.1 | 16.8 | 9.6 | 20.8 | 3.4 | 2.3 |
| 2010 | China | 77 | 0.4 | 1.9 | 1.8 | 19 | 0 |
|  | Japan | 27.1 | 7.8 | 28.4 | 24.6 | 10.4 | 1.8 |
|  | S. Korea | 46.4 | 4 | 16.4 | 31.3 | 1.9 | 0.1 |
|  | Taiwan | 49.5 | 4.5 | 24.4 | 16.8 | 3.5 | 1.2 |
| 2020 | China | 63.5 | 0.1 | 3 | 4.7 | 28.7 | 0 |
|  | Japan | 30.6 | 3.1 | 38.8 | 3.8 | 22.1 | 1.7 |
|  | S. Korea | 34.8 | 1.3 | 28.8 | 27.9 | 6.6 | 0.6 |
|  | Taiwan | 45 | 1.6 | 35.7 | 11.2 | 5.4 | 1.1 |
| 2030 | China | 51 | 0 | 0 | 10 | 40 | 0 |
|  | Japan | 19 | 2 | 20 | 20-22 | 36-38 | 1 |
|  | S. Korea | 19.7 | 0 | 22.9 | 32.8 | 21.6 | 3 |
|  | Taiwan | 30 | 0 | 50 | 0 | 20 | 0 |

**2. Robustness check: hierarchical cluster analysis using alternative outcome metrics**

**Methods**

To test whether our six-cluster typology varies based on the specific climate-performance indicators used, we repeated the entire Ward-linkage hierarchical clustering workflow after replacing the four CCPI subscores with the four more technically oriented SDG-7/SDG-13 measures (CO₂-intensity of power, renewable-energy share, territorial fossil-fuel emissions, and emissions embodied in trade; see Table S1). All interest-, institution-, and idea-side variables were left unchanged and, as before, were z-standardised prior to analysis.

The NbClust diagnostics applied to the SDG data pointed to either *k = 2* or *k = 10* as acceptable cut points; we adopted *k = 10* to retain interpretive nuance. For direct comparison we then generated a ten-cluster solution in place of the original six-cluster CCPI dendrogram using the same parameters.

**S2 Table 2. Variables used for robustness analysis.**

| **Theoretical Variable** | **Empirical Variable** | **Description** | **Year of Data Collection** |
| --- | --- | --- | --- |
| **Dependent Variables** |  |  |  |
| GhG Emissions | GhGs_CCPI1 | CCPI Scale, 0-100 (high is positive) | 2021 |
| Renewable | Renewables_CCPI2 | CCPI Scale, 0-100 | 2021 |
| CO2 Intensity | EnergyUse_CCPI3 | CCPI Scale, 0-100 | 2021 |
| Climate Policies | ClimatePolicy_CCPI4 | CCPI Scale, 0-100 | 2021 |
|  |  |  |  |
| CO2 Intensity (CO₂ emissions from fuel combustion per total electricity output (MtCO₂/TWh) | sdg7_co2twh | SDG Scale, 0-100 (high is positive) | 2022 |
| Renewable energy share in total final energy consumption (%) | sdg7_renewcon | SDG Scale, 0-100 (high is positive) | 2022 |
| CO₂ emissions from fossil fuel combustion and cement production (tCO2/capita) | sdg13_co2gcp | SDG Scale, 0-100 (high is positive) | 2022 |
| GHG emissions embodied in imports (tCO₂/capita) | sdg13_ghgimport | SDG Scale, 0-100 (high indicates greater imports of embodied GHGs) | 2022 |
|  |  |  |  |

**Results**

Spearman correlations between matched CCPI and SDG indicators (Fig S1; median ρ = 0.72, all *p* < 0.01) confirmed that the two indicator sets capture closely related transition dynamics while differing in their emphasis on forward-looking policy versus realised emissions. The one seemingly incongruous result is the negative correlation between two measures of carbon intensity (sdg7_co2twh and EnergyUse_CCPI3). The likely causes is that the CCPI measures indicates progress or improvement in carbon intensity, while the SDG measures is an absolute measure.


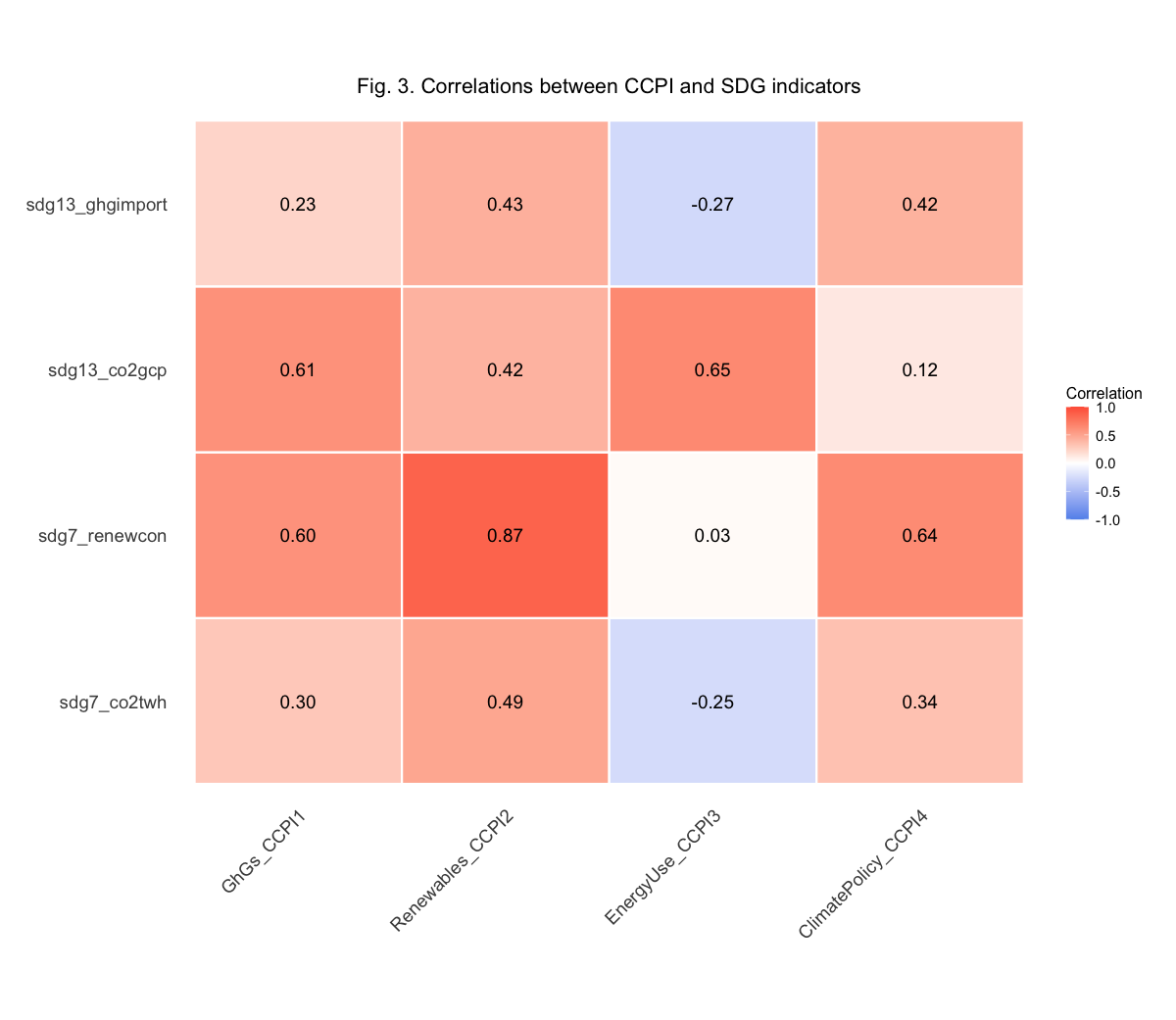


**S2 Fig 1. Spearman correlations between CCPI variables and SDG 7 and SDG 13 variables**

The ten-cluster CCPI solution (Fig S2) is essentially a nested refinement of the six-cluster tree in the main study: every original branch re-appears intact, merely splitting into one to three sub-branches (e.g., the “Scandinavia” cluster divides Sweden from its Nordic peers, while “East Asia” separates China from Japan–Korea–Taiwan). No country crosses the six top-level boundaries, and average silhouette widths remain above 0.30, indicating well-defined groups. The SDG-based dendrogram mirrors the CCPI-10 tree: 24 of 28 countries fall into the same ten branches, and the characteristic macro-clusters—Scandinavia, Western democracies, East Asian developmental states, coal-heavy emerging economies, along with the single cases of India and Russia—re-emerge unchanged. The few divergences, such as South Africa joining China when policy scores are removed, are consistent with the SDG indicators’ heavier weighting of absolute emissions. In short, using the non-policy focused SDG outcome variables and allowing the algorithm to form finer partitions leaves the overarching “architectures of constraint” untouched, reinforcing the substantive validity of the six-cluster typology presented in the main text.

| **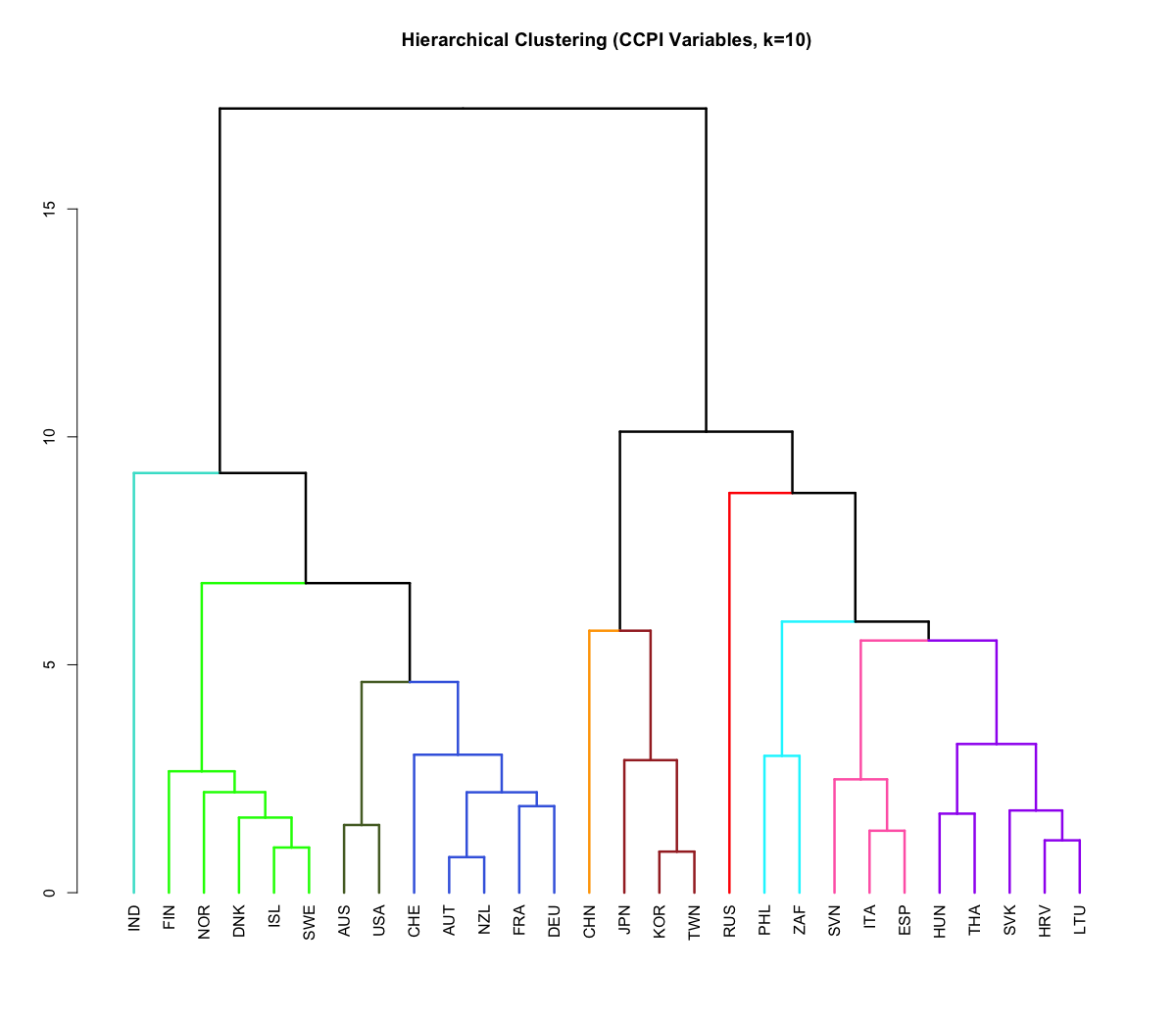** |
| --- |

**Fig S2. Dendrograms showing ten clusters solutions for CCPI variables**

| **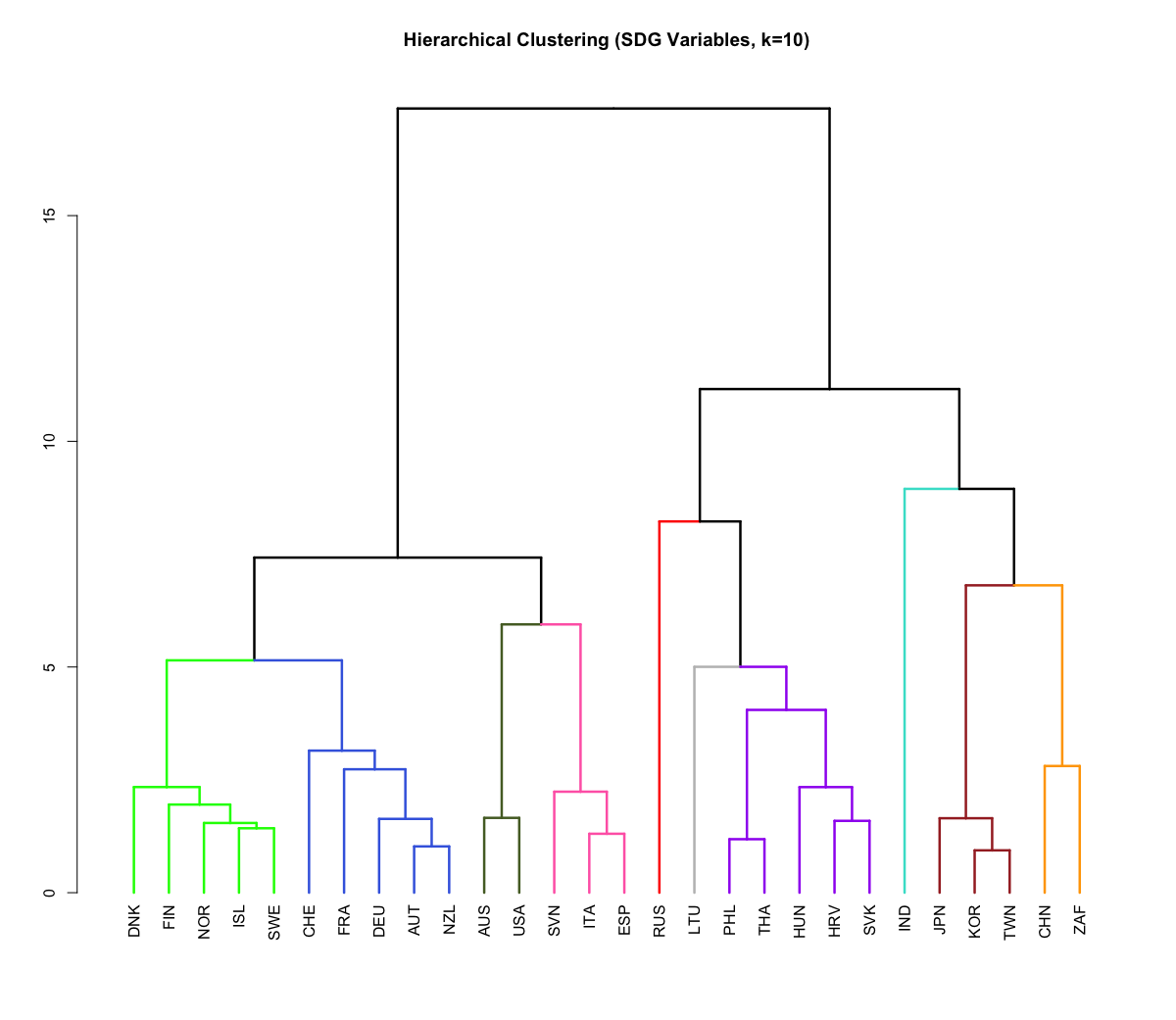** |
| --- |

**Fig S3. Dendrograms showing ten clusters solutions for SDG variables**
